# Species-specific transcriptional profiles of the gut and gut microbiome of *Ceratitis quilicii* and *Ceratitis rosa sensu stricto*

**DOI:** 10.1038/s41598-019-54989-z

**Published:** 2019-12-04

**Authors:** Fathiya M. Khamis, Paul O. Mireji, Fidelis L. O. Ombura, Anna R. Malacrida, Erick O. Awuoche, Martin Rono, Samira A. Mohamed, Chrysantus M. Tanga, Sunday Ekesi

**Affiliations:** 1 International Centre of Insect Physiology and Ecology, P.O. Box 30772-00100, Nairobi, Kenya; 2 Biotechnology Research Institute, Kenya Agricultural and Livestock Research Organization, P.O. Box 362-00902, Kikuyu, Kenya; 3 Centre for Geographic Medicine Research Coast, Kenya Medical Research Institute, P.O. Box 428, Kilifi, Kenya; 4 Department of Agriculture, School of Agriculture and Food Science, Meru University of Science and Technology, P.O. Box 972, Meru, Kenya; 5 Department of Biology and Biotechnology, Universita degli Studi di Pavia, Corso Strada Nuova, 65, 27100 Pavia, Italy

**Keywords:** Molecular biology, Transcriptomics

## Abstract

The fruit fly species, *Ceratitis rosa sensu stricto* and *Ceratitis quilicii*, are sibling species restricted to the lowland and highland regions, respectively. Until recently, these sibling species were considered as allopatric populations of *C. rosa* with distinct bionomics. We used deep Next Generation Sequencing (NGS) technology on intact guts of individuals from the two sibling species to compare their transcriptional profiles and simultaneously understand gut microbiome and host molecular processes and identify distinguishing genetic differences between the two species. Since the genomes of both species had not been published previously, the transcriptomes were assembled *de novo* into transcripts. Microbe-specific transcript orthologs were separated from the assembly by filtering searches of the transcripts against microbe databases using OrthoMCL. We then used differential expression analysis of host-specific transcripts (i.e. those remaining after the microbe-specific transcripts had been removed) and microbe-specific transcripts from the two-sibling species to identify defining species-specific transcripts that were present in only one fruit fly species or the other, but not in both. In *C. quilicii* females, bacterial transcripts of *Pectobacterium spp*., *Enterobacterium buttiauxella, Enterobacter cloacae* and *Klebsiella variicola* were upregulated compared to the *C. rosa s.s*. females. Comparison of expression levels of the host transcripts revealed a heavier investment by *C. quilicii* (compared with *C. rosa s.s*.) in: immunity; energy production; cell proliferation; insecticide resistance; reproduction and proliferation; and redox reactions that are usually associated with responses to stress and degradation of fruit metabolites.

## Introduction

The Natal fruit fly, *Ceratitis rosa* Karsch, is a polyphagous Afro-tropical pest species with a host range of over 100 wild and cultivated plants^1^. In Africa, *C. rosa* is found in southern and eastern Africa^2,3^, including the Indian Ocean islands of Mauritius and La Réunion, where it is an invasive and devastating quarantine pest^4–6^. *Ceratitis rosa* occurs in R1 and R2 morphotypes^7^ which have recently been described as the sibling species *C. rosa sensu stricto* and *Ceratitis quilicii*^8^. In Kenya, *C. rosa* is confined to coastal areas^2,3^ while *C. quilicii* is confined to central highland regions (1,533–1,771 m above sea level)^9^. The spatial geographic segregation of these sibling species appears to be defined by differential thermal developmental requirements of the two species^6,10,11^.

Physiologically, the midgut of organisms, including *C. rosa sensu lato* represents the physiological contact between the organism and its environment; it is possible that microbial (gut microbiome) and host transcript expression profiles of the midgut would be informed by the environments in which these flies exist. Despite our limited knowledge of the gut microbiome of these insects^12^, available information suggests a wide range of insect-microbe interactions (from symbionts to facultative bacteria) in nature^13^. While symbionts and their hosts are interdependent, vertically or environmentally-acquired facultative bacteria^14^ are not essential for host survival, but can influence host fitness and ecological adaptation to the environment^15^. Amongst fruit flies, the gut microbiome of *Ceratitis capitata* is largely dominated by free-living *Pectobacterium*, *Enterobacter* and *Klebsiella* species from the Enterobacteriaceae family, that are stable throughout the life cycle of the fly and between geographical regions^16–20^. Unlike vertically-transmitted endosymbionts, these extracellular bacteria are transmitted horizontally^21^, and have only been shown recently to have clear impact on the germ line and reproduction via modulation of oogenesis, maternal-to-zygotic-transition in offspring and phenotypic variation in mutants mediated by fly molecular factors^22^. Many chronic infectious agents have subtle deleterious effects on hosts^23^. Also, there is potential for host transcriptional profiles to be influenced by the gut microbiome as well as other environmental factors, which can thus define species-specific adaptation to the local environment. The recent delineation of the *C. rosa* morphotypes into distinct sibling species necessitates further characterisation of their genotypes to identify inherent molecular differences in transcripts of both the gut microbiome and the host that will allow the two morphologically identical species to be distinguished from each other.

In this study, we conducted deep Next Generation Sequencing (NGS) of intact guts from the sibling species, *C. rosa s.s* and *C. quilicii*. We isolated host and microbial transcripts from the transcriptome and identified microbe-specific and host-specific transcripts that were differentially expressed in the two species.

## Results

### *De novo* assembly and mapping statistics of the transcriptome from *C. rosa s.s* and *C. quilicii* transcripts

**Figure 1:**
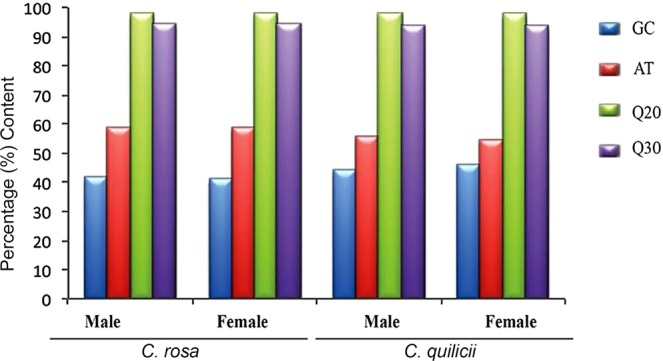
Quality matrices of the *de novo* assembly of RNA-seq reads using the short reads Trinity assembly program^24,39^. GC = Guanine-Cytosine content, AT = Adenine-Thymine content, Q20 = PHRED quality score threshold of 20, Q30 = PHRED quality score threshold of 30.

**Figure 2:**
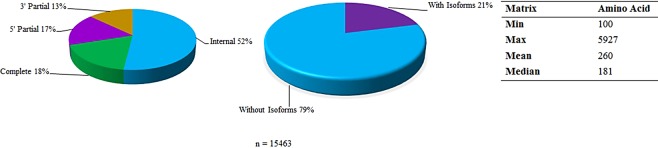
Nature of open reading frames (ORFs) isolated from the assembly using the TransDecoder program^24^.

**Figure 3:**
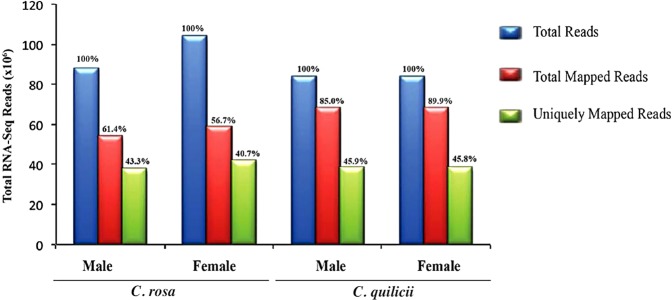
Mapping statistics of the RNA-seq reads to transcripts.

High quality (83–103 million) reads were obtained with at least 93.46 and 41.49% Q30 and GC contents from *C. rosa s.s*. and *C. quilicii* libraries (male and female combined), respectively (Fig. 1). We successfully assembled the reads, *de novo*, into 36170 transcripts with an overall N100, median and average length of 738, 336 and 552 nucleotides, respectively. Our TransDecoder ORF analysis^24^ identified 15463 transcripts as the best candidates (with eclipsed ORFs removed) (Fig. 2). Most of the ORFs were internal Coding Domain Sequences (CDS) without isoforms. Complete CDs constituted about 18% of the transcripts (Fig. 2). Approximately 41.0–71.3% of the reads were successfully mapped to our transcripts and at least 43.6% of our read mappings were unique to specific transcripts (Fig. 3).

### OrthoMCL separation of host and microbiome transcripts

**Figure 4:**
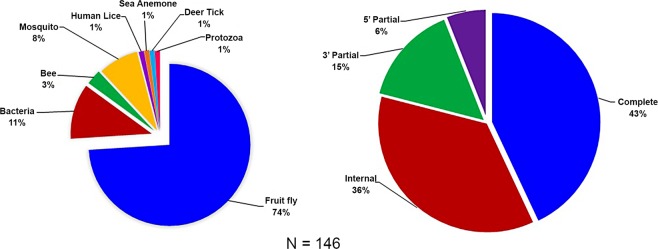
Proportional representation of taxa that had at least 98% sequence identity and matched specific *C. rosa* transcripts in the orthoMCL database^26^; and proportional assembly of their *de novo* ORFs using the short reads Trinity assembly program^24,39^.

**Figure 5:**
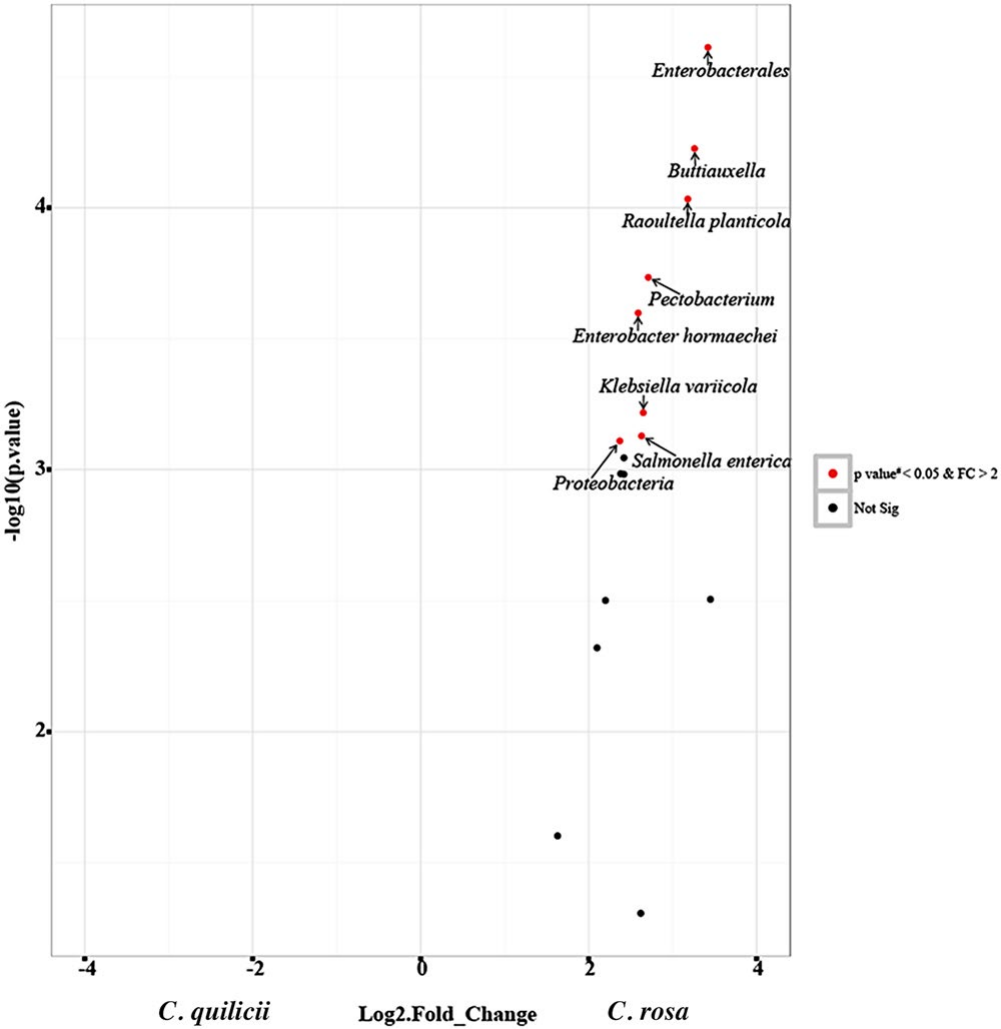
Volcano plot of RNA-Seq of bacterial transcripts that were differentially expressed in female *C. rosa s.s* and *C. quilicii* and used as a proxy measurement of relative abundance of the respective taxa.

When results from the OrthoMCL analysis^25^ of the *de novo* assembled gut transcripts were mapped against the orthoMCL database^26^ var 5 it was revealed that most of our gut transcripts had fruit fly orthologs with fewer orthologs from bacteria; most orthologs were complete CDS (Fig. 4). On further analysis, 72.3% of the host transcripts were homologous to *C*. *capitata* fruit flies (Table 1) while the bacterial transcripts were homologous to 13 species of bacteria (Table 2). RNA-Seq differential analysis amongst the bacterial transcripts from *C. rosa s.s* and *C. quilicii* and from male vs. female flies revealed significant upregulation of transcripts associated with eight species of bacteria in female *C. quilicii* compared with corresponding *C. rosa s.s* females (Fig. 5). Induction of bacteria-associated transcripts was similar amongst the remaining comparison categories (P > 0.05).

**Table 1:** Eukaryotic taxa identified in the orthoMCL database^26^, that had orthologs in the *C. rosa s.s*. and *C. quilicii* transcripts, and their corresponding homologs in the nr NCBI database.

OrthoMCL Orthologs*		*Ceratitis rosa s.s*. and *Ceratitis quilicii* transcripts		NCBI BLAST homologs**	
Best BLAST Hits				Best BLAST Hits (nr database)	
**Taxon**	**E- value**	**N**	**Length (aa)**	**Species**	**E- value**
*Aedes aegypti*	5E-92	1	161	*Bactrocera dorsalis*	8E-112
	6E-58	1	103	*Bactrocera oleae*	4E-52
	1E-105 -1E-71	2	129–183	*Drosophila melanogaster*	2E-87 - 5E-130
	1E-147	1	248	*Homalodisca liturata*	1E-170
*Anopheles gambiae*	8E-56	1	100	*Cuerna arida*	2E-56
	1E-64	1	112	*Monomorium pharaonis*	3E-77
*Apis*	0E + 00	1	377	*Ceratitis capitata*	0
	5E-85	2	148	*Drosophila melanogaster*	2E-104
	6E-57	1	101	*Operophtera brumata*	4E-62
	4E-82	1	150	*Trichinella pseudospiralis*	1E-101
*Culex pipiens*	0E + 00	3	100–493	*Ceratitis capitata*	0 - 8E - 60
	2E-85	1	141	*Drosophila affinis*	5E-98
	0E + 00	1	304	*Drosophila melanogaster*	0
*Drosophila melanogaster*	1E-103	1	181	*Drosophila pseudoobscura*	3E-128
	7E-74	1	128	*Anopheles gambiae*	4E-87
	2E-66	1	124	*Drosophila biarmipes*	5E-59
	3E-58	1	109	*Drosophila elegans*	6E-69
	1E-64	1	117	*Drosophila erecta*	7E-71
	3E-98	1	155	*Drosophila ficusphila*	5E-117
	6E-88	1	148	*Drosophila miranda*	4E-102
	0	1	329	*Drosophila montana*	0
	3E-59	1	108	*Drosophila navojoa*	2E-65
	2E-72	1	129	*Drosophila suzukii*	5E-88
	6E-83	1	144	*Drosophila virilis*	1E-97
	1E-180 - 2E-58	2	102–305	*Drosophila busckii*	0 - 2E-69
	1E-86 - 7E-71	2	120–156	*Drosophila persimilis*	8E-105 - 2E-80
	1E-111-2E-99	2	173–191	*Drosophila willistoni*	1E-115- 3E-138
	5E-82 - 6E-68	3	106–139	*Drosophila simulans*	8E-94 - 2E-79
	1E-55 - 1E-64	3	102–113	*Musca domestica*	4E-75 - 2E-67
	0 - 6E-82	5	116–606	*Bactrocera oleae*	0 - 3E-101
	0 - 1E-150	6	101–479	*Bactrocera dorsalis*	0 - 1E-168
	0 - 1E-59	7	105–586	*Bactrocera cucurbitae*	0 - 5E-113
	0 - 3E-93	7	161–381	*Rhagoletis zephyria*	0 - 3E-121
	0 - 1E-112	7	184	*Rhagoletis zephyria*	0 - 1E-124
	0 - 1E-52	10	105–409	*Drosophila melanogaster*	0 - 1E-55
	0 - 7E-54	50	100–1046	*Ceratitis capitata*	0 - 5E-60
*Ixodes scapularis*	8E-75	1	137	*Felis catus*	1E-91
	4E-74	1	141	*Nothobranchius furzeri*	7E-91
*Nematostella vectensis*	5E-76	1	137	*Culex quinquefasciatus*	4E-92
*Pediculus humanus*	3E-61	1	111	*Ceratitis capitata*	3E-75
*Tetrahymena thermophila*	3E-56	1	104	*Acipenser persicus*	4E-67

* = Orthologs of *C. rosa s.s*. and *C. quilicii* transcripts with at least 98% identity with matches in the orthoMCL database.

** = Species in the nr NCBI database with genes orthologous to the *C. rosa s.s*. and *C. quilicii* orthologs in the orthoMCL database, and with at least 98% sequence identity and coverage (without gaps) in the NCBI database.

**Table 2:** Bacterial taxa identified in the orthoMCL database^26^, with orthologs in the *C. rosa s.s*. and *C. quilicii* transcripts, and their corresponding homologs in the NCBI nr database.

OrthoMCL Orthologs*		*Ceratitis rosa transcripts*		NCBI BLAST homologs**	
Best BLAST Hits				Best BLAST Hits (nr database)	
Taxon	E- value	ID	Length (aa)	Species	E- value
*Escherichia coli*	7E-67	C.r_14811	125	*Buttiauxella*	3E-82
	1E-90	C.r_8435	161	*Pectobacterium*	5E-97
	1E-105	C.r_8944	187	*Enterobacteriaceae*	9E-132
*Salmonella enterica*	9E-58	C.r_14824	106	*Salmonella enterica*	2E-68
*Salmonella enterica*	5E-73	C.r_16126	131	*Enterobacter cloacae* complex	3E-89
	1E-56	C.r_16175	143	*Klebsiella variicola*	9E-70
	1E-79	C.r_16852	141	*Raoultella planticola*	9E-95
	1E-110	C.r_17300	186	*Salmonella enterica*	2E-131
	1E-107	C.r_4220	190	*Escherichia coli*	2E-132
	7E-76	C.r_4222	136	*Enterobacter cloacae*	6E-90
	2E-66	C.r_6036	124	*Enterobacterales*	4E-81
	2E-60	C.r_6303	112	*Escherichia coli*	3E-73
*Shigella flexneri*	7E-66	C.r_6038	124	*Enterobacterales*	3E-81
	1E-117	C.r_7072	210	*Proteobacteria*	8E-146
	4E-71	C.r_7640	126	*Enterobacter cloacae*	3E-85
*Yersinia enterocolitica*	1E-167	C.r_8942	290	*Enterobacter hormaechei*	0

* = Orthologs of *C. rosa s.s*. and *C. quilicii* transcripts with at least 98% identity with matches in the orthoMCL database.

** = Species in the nr NCBI database with genes orthologous to the *C. rosa s.s*. and *C. quilicii* orthologs in the orthoMCL database, and with at least 98% sequence identity and coverage (without gaps) in the NCBI database.

### Differential expression of host gut transcripts between male and female *C. rosa s.s*. and *C. quilicii* libraries

**Figure 6:**
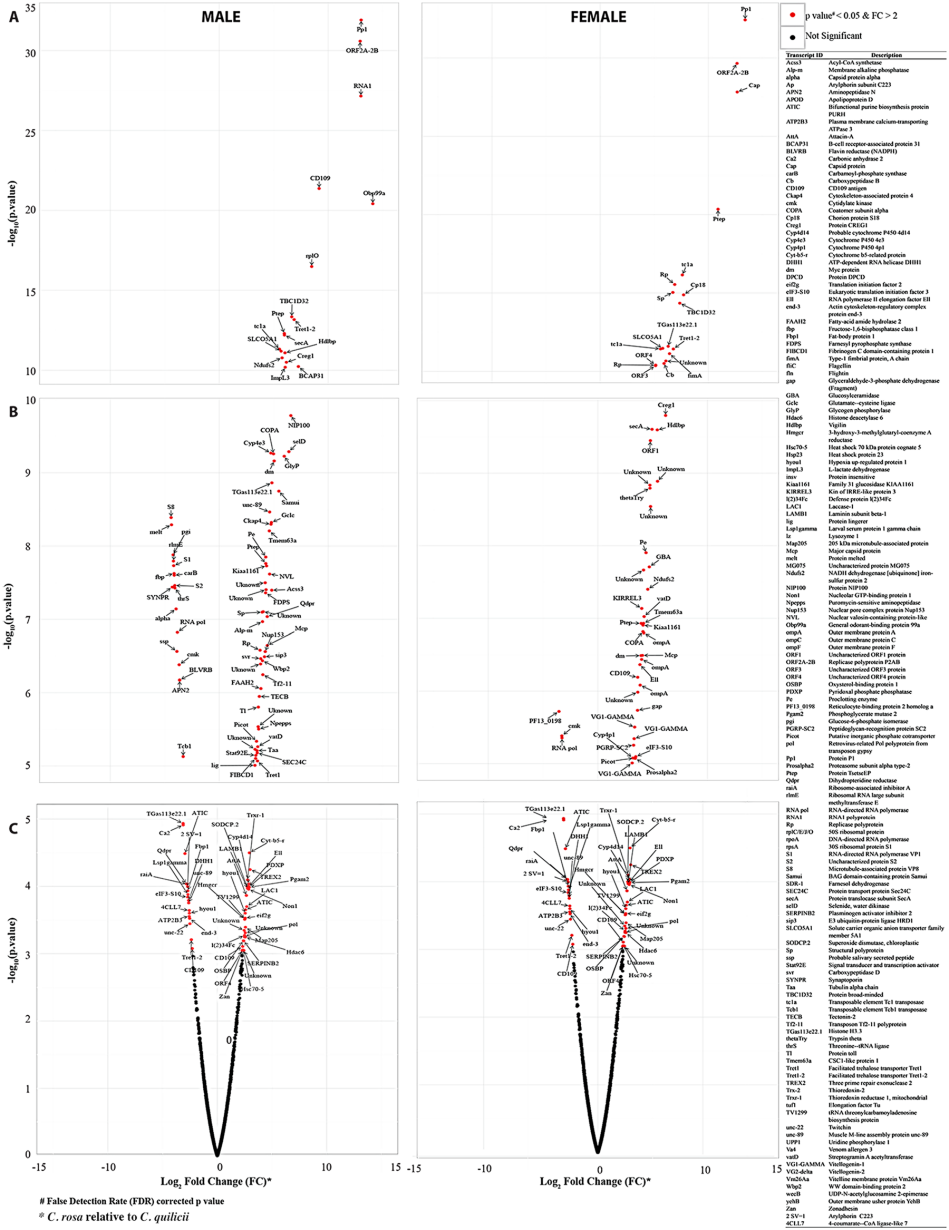
Volcano plot showing host transcripts that were differentially expressed in the gut tissues of *C. rosa s.s* and *C. quilicii*. The transcripts expression level data were supported by at least 100 reads in each category (i.e. in *C. rosa s.s* or *C. quilicii*) or five CPM (Counts Per Million) by edge R analysis^44,45^. The red dots indicate points-of-interest, i.e. the best BLAST hits^53^ on the manually annotated and reviewed Swiss-Prot database^54^, that displayed both large magnitude fold-changes (x axis) and high statistical significance (−log10 of p value, y axis). Points with a fold-change less than 2 (log_2_ = 1) and/ or a False Detection Rate (FDR) corrected p value of less than 0.05 are shown in black, and indicate transcripts that did not change significantly in expression between the two species. Panel A, B, C = the transcripts that had the most, medium and least difference in their expression between the two species.

RNA-Seq differential analysis of host gut-specific transcripts revealed High proportion of significantly induced host transcripts in *C. quilicii* compared with *C. rosa s.s* for both genders (Fig. 6). In male *C. quilicii* (compared with male *C. rosa s.s*.), GSEA GO term enrichment analysis^27–29^ revealed significant induction of putative pathways associated with: innate immune responses against microbes; apoptosis-related cellular stress responses linked to endoplasmic reticulum (ER); negative regulation of immunity; catalytic transfer of hexose sugar across membranes; and intra–organismal carbohydrate catabolism (Table 3). Further to this, STRING database enriched pathway analysis^30^ revealed induction of putative pathways associated with: phagocytosis; ER protein processing; glycolysis; folate biosynthesis (crucial for DNA replication and cell division); plant terpenoids; and limonene and pinene degradation (Table 4). Volcano plot analysis of individual gene sequences associated with these observations identified further transcripts that were upregulated in male *C. quilicii* compared to male *C. rosa s.s* (Fig. 6) and that the majority of these were primarily associated with: energy production (mitochondrial Acyl-CoA synthetase, glycogen phosphorylase, L-lactate dehydrogenase, glucosidase, NADH dehydrogenase and nuclear valosin); immunity (CD109, protein TsetseEP, proclotting enzyme antigen, tectonin-2, toll, streptogramin A acetyltransferase); cell growth and proliferations (carboxypeptidase D, signal transducer and transcription activator, transposon, selenide, replication polyprotein, BAG domain-containing protein samui, myc, replicase polyprotein, and protein P1); synthesis and transport of protein and other macromolecules (solute carrier organic anion transporter, RNA1, polyprotein, 50 S ribosomal protein, protein translocase, odorant-binding protein, alkaline phosphatase, B-cell receptor-associated protein, nuclear pore complex protein, inorganic phosphate cotransporter, trehalose transporter and protein transport protein); and resistance to insecticides (cytochrome P450). Only transcripts of pathways associated with urine nucleotide biosynthesis (major energy carriers) were more highly expressed in male *C. rosa s*.s. compared with male *C. quilicii* (Tables 3 and 4). Volcano plot analysis also revealed that most of the transcripts that were more highly expressed in male *C. rosa s.s* were of viral (capsid protein alpha, RNA-directed RNA polymerase, threonine-tRNA ligase and microtubule-associated protein) or bacterial (cytidylate kinase) origin. The rest of the transcripts induced in male *C. rosa s.s* were largely associated with energy metabolism (NADPH, carbamoyl-phosphate synthase, protein melted, fructose-1,6-bisphosphatase).

**Table 3:** Canonical gene set enrichment analysis (GSEA) of transcripts that were significantly differentially expressed transcripts in the gut tissues of either male or female *C. rosa s.s* and *C. quilicii*. Enrichment profiles established using the WEB-based GEne SeT AnaLysis Toolkit (WebGestalt)^46^. Non-redundant enriched Gene Ontology (GO) categories.

Species	Category	Pathway ID	Description of Pathway	#Ref	#Observed	Expected	Ratio	P-Value	Adjusted P-Value
*Ceratitis quilicii* Male	Biological process	GO:0019730	Antimicrobial humoral response	106	5	0.64	7.81	0.0004	4.93E-02
		GO:0030968	Endoplasmic reticulum unfolded protein response	8	2	0.05	41.41	0.001	4.93E-02
		GO:0015149	Hexose transmembrane transporter activity	18	2	0.11	18.1	0.0053	1.44E-01
		GO:0050777	Negative regulation of immune response	11	2	0.07	30.11	0.0019	4.93E-02
		GO:0044724	Single-organism carbohydrate catabolic process	39	3	0.24	12.74	0.0016	4.93E-02
	Molecular function	GO:0042803	Protein homodimerization activity	72	3	0.44	6.79	0.0097	1.44E-01
	Cellular component	GO:0030662	Coated vesicle membrane	23	2	0.15	13.67	0.0092	6.58E-02
		GO:0009897	External side of plasma membrane	8	2	0.05	39.3	0.0011	2.69E-02
		GO:0005811	Lipid particle	249	7	1.58	4.42	0.0009	2.69E-02
		GO:0045298	Tubulin complex	10	2	0.06	31.44	0.0017	2.69E-02
		GO:0030018	Z disc	21	2	0.13	14.97	0.0077	6.58E-02
*Ceratitis rosa* Male	Biological process	GO:0006164	Purine nucleotide biosynthetic process	72	2	0.11	17.48	0.0056	2.37E-01
	Molecular function	GO:0000166	Nucleotide binding	1225	3	1.91	1.57	0.2973	5.79E-01
		GO:0045735	Nutrient reservoir activity	5	2	0.01	256.29	2.27E-05	5.00E-04
		GO:0016491	Oxidoreductase activity	630	2	0.98	2.03	0.2577	5.79E-01
		GO:0022891	Substrate-specific transmembrane transporter activity	649	2	1.01	1.97	0.269	5.79E-01
		GO:0008270	Zinc ion binding	875	2	1.37	1.46	0.4023	5.79E-01
	Cellular component	GO:0005616	Larval serum protein complex	5	3	0.01	437.29	1.92E-08	3.84E-07
		GO:0005811	Lipid particle	249	3	0.34	8.78	0.0041	1.64E-02
		GO:0005886	Plasma membrane	603	2	0.83	2.42	0.1983	4.67E-01
*Ceratitis quilicii* Female	Biological process	GO:0045454	Cell redox homeostasis	50	2	0.23	8.78	0.0216	4.67E-01
		GO:0008340	Determination of adult lifespan	128	3	0.58	5.15	0.0203	4.67E-01
		GO:0008593	Regulation of Notch signalling pathway	62	2	0.28	7.08	0.0322	5.51E-01
		GO:0001666	Response to hypoxia	45	2	0.2	9.76	0.0177	4.49E-01
		GO:0007296	Vitellogenesis	8	2	0.04	54.89	0.0006	1.12E-01
	Molecular function	GO:0015036	Disulfide oxidoreductase activity	30	2	0.13	14.9	0.0079	7.24E-02
		GO:0009055	Electron carrier activity	145	3	0.65	4.62	0.0267	1.47E-01
		GO:0051287	NAD binding	39	2	0.17	11.46	0.013	1.02E-01
		GO:0050661	NADP binding	12	2	0.05	37.25	0.0013	7.15E-02
		GO:0016651	Oxidoreductase activity, acting on NADH or NADPH	51	2	0.23	8.77	0.0217	1.33E-01
		GO:0005198	Structural molecule activity	487	7	2.18	3.21	0.0054	7.24E-02
	Cellular component	GO:0005576	Extracellular region	814	7	3.86	1.81	0.0847	4.49E-01
		GO:0015934	Large ribosomal subunit	102	2	0.48	4.14	0.0839	4.49E-01
		GO:0005811	Lipid particle	249	7	1.18	5.93	0.0001	5.30E-03
		GO:0005875	Microtubule associated complex	362	5	1.72	2.91	0.0269	4.49E-01
		GO:0005700	Polytene chromosome	121	3	0.57	5.23	0.0193	4.49E-01
*Ceratitis rosa* Female	Biological process	GO:030154	Cell differentiation	1799	2	0.76	2.62	0.1664	3.78E-01
	Molecular function	GO:0045735	Nutrient reservoir activity	5	2	0	961.1	1.30E-06	2.60E-06
	Cellular component	GO:0005616	Larval serum protein complex	5	3	0	962.04	1.16E-09	1.62E-08
		GO:0005811	Lipid particle	249	3	0.16	19.32	0.0003	2.10E-03

**Table 4:** Enriched KEGG pathways of transcripts that were significantly differentially expressed transcripts in the gut tissues of either male or female *C. rosa s.s*. and *C. quilicii*. Enrichment profiles established using the WEB-based GEne SeT AnaLysis Toolkit (WebGestalt)^46^.

Species	Pathway Name	#Ref	#Observed	Expected	Ratio	P-Value	Adjusted P-Value
*Ceratitis quilicii* Male	Metabolic pathways	892	12	4.3	2.79	0.001	0.0076
	Phagosome	64	2	0.31	6.49	0.0381	0.0478
	Propanoate metabolism	22	2	0.11	18.87	0.005	0.009
	Protein processing in endoplasmic reticulum	119	4	0.57	6.98	0.0026	0.0078
	Ribosome biogenesis in eukaryotes	78	2	0.38	5.32	0.0543	0.0543
	Terpenoid backbone biosynthesis	13	2	0.06	31.93	0.0017	0.0076
	Folate biosynthesis	21	2	0.1	19.77	0.0045	0.009
	Glycolysis/Gluconeogenesis	49	2	0.24	8.47	0.0232	0.0348
	Limonene and pinene degradation	68	2	0.33	6.1	0.0425	0.0478
*Ceratitis rosa* Male	Metabolic pathways	892	3	1.15	2.6	0.1046	0.1046
*Ceratitis quilicii* Female	Limonene and pinene degradation	68	2	0.23	8.7	0.0221	0.0418
	Metabolic pathways	892	6	3.01	1.99	0.0783	0.0783
	Pyrimidine metabolism	77	2	0.26	7.69	0.0279	0.0418
*Ceratitis rosa* Female	-	—	—	—	—	—	—

**#Ref**: the number of reference genes in the category; **# Observed**: the number of genes in the gene set and in the category; **Expected**: the expected number in the category; **Ratio**: ratio of enrichment; **P-Value**: p value from hypergeometric multiple Test Adjustment test; **Adjusted P-value**: p value adjusted by the multiple test adjustment.

When female *C. rosa s.s* and *C. quilicii* were compared, the pathways induced in *C. quilicii* females compared with *C. rosa s.s* females were associated with: yolk formation; maintenance of the cellular redox environment; control of viability and duration of the adult phase of the life cycle; and regulation of notch signaling pathways that affect cell differentiation and responses to insufficient oxygen (hypoxia) (Fig. 6). In addition to the upregulation of the general metabolic pathways, and limonene and pinene degradation pathways that were observed in male *C. quilicii*, pyrimidine metabolism pathways were also upregulated in female *C. quilicii* (Table 4). Analysis of the volcano plot of individual gene expression profiles (Fig. 6) of female *C. quilicii* (compared with female *C. rosa s.s*.) revealed upregulation of pathways associated with: some common energy production regulators (glucosidase and NADH dehydrogenase); immunity (CD109, protein TsetseEP, proclotting enzyme antigen and streptogramin A acetyltransferase); cell growth and proliferations (replication polyprotein, myc, replicase polyprotein, and protein P1); synthesis and transport of protein and other macromolecules (solute carrier organic anion transporter, protein translocase and inorganic phosphate cotransporter and trehalose transporter); and resistance to insecticide (cytochrome P450). Transcripts that were more highly expressed in female *C. rosa s.s* (compared with female *C. quilicii*) included pathways associated with: energy metabolism (glyceraldehyde-3-phosphate and glucosylceramidase dehydrogenase); immunity (peptidoglycan-recognition protein); transcription (eukaryotic translation initiation factor, RNA polymerase and coatomer); proteolysis (trypsin theta, proteolysis); and egg formation/reproduction (chorion protein, outer membrane protein A, vitellogenin and vigilin). Only transcripts for pathways associated with urine nucleotides biosynthesis (major energy carriers) were more highly expressed in male *C. rosa s.s* compared with male *C. quilicii* (Table 4). Volcano plot analysis revealed that most of the transcripts that were more highly expressed in male *C. rosa s.s*. were of viral (capsid protein alpha, RNA-directed RNA polymerase, threonine-tRNA ligase and microtubule-associated protein) or bacterial (cytidylate kinase) origin. The rest were associated with energy metabolism (NADPH, carbamoyl-phosphate synthase, protein melted, fructose-1,6-bisphosphatase). Up-regulation of bacteria/virus-specific (cytidylate kinase and RNA-directed RNA polymerase) transcripts was also common in female *C. rosa s.s*. The comparison made in this study of expression profiles of 11 randomly selected DE genes from male and female datasets, revealed a Pearson correlation coefficient of R = 0.8146 and R = 0.9338 (Text S1) for the genes evaluated, which is indicative of a valid transcriptome.

## Discussion

The goals of this study were (1) to establish the identity of naturally-occurring gut microbes (gut microbiome) associated with the sibling species, *C. rosa s.s*. and *C. quilicii* and (2) to use host transcriptional profiles to identify host species-specific transcripts that define the identity of *C. rosa s.s*. and *C. quilicii* sibling species. The *C. rosa s.s*. (ex Kibarani, Msambweni district) and *C*. *quilicii* (ex Kithoka, Imenti North district) colonies used in this study were reared on carrot-based artificial diets with no antibiotics. Oviposition was done on apple mango domes and the insects reared in rooms with similar conditions of their places of origin (28 ± 1 °C, 50 ± 8% RH for *C. rosa s.s*. and 23 ± 1 °C, 65 ± 5% RH for *C*. *quilicii*). This was essential so as to minimize the effects of environmental differences on the colonies. Ideally, sample collections were to be of similar generation age, but we realized that the two colonies had different reproductive rates. The fecundity rate of *C. quilicii* was way higher than that of *C. rosa s.s*. hence *C. quilicii* colony was at the 59^th^ generation while the *C. rosa s.s*. colony was at the 36^th^ generation.

Using OrthoMCL analyses we identified, transcripts of 13 distinct extracellular bacterial species, some of which (*Pectobacterium*, *Enterobacter* and *Klebsiella* species from the family *Enterobacteriaceae*) have previously been identified as stable free-living bacteria dominating the gut microbiome of *C. capitata* across its geographical distribution^16–20^. Others like *Enterobacterium buttauxella* are known to proliferate in a stable way throughout the life cycle of *C. capitata*^31^. Surprisingly, we also detected phytopathogenic *Pectobacterium* spp. in *C. quilicii*, which may have colonized and ultimately evolved alternative associations, perhaps involving insect mediation of plant pathogen dissemination^32^. Another plausible explanation would be it could have originated from the carrot-based diet since the diet was made devoid of antibiotics. Kahala *et al*.^55^ demonstrated that carrots infected with this bacterium appeared mainly in shop samples during autumn, winter, and spring, causing spoilage gradually. Others, such as *Enterobacter cloacae* and *Klebsiella variicola* have also been reported colonizing the gut of *C. capitata* and *Zeugodacus cucurbitae* in nature^33,34^. The reason for the presence of some bacterial species in *C. quilicii*, and not in *C. rosa s.s*, and the reverse scenario for the presence of virus transcripts in *C. rosa s.s*. and not *C. quillicii*, is not obvious but may reflect the prevalence of these bacteria/virus species in the original environments of the two species, respectively. It may also relate to the original host plant from which the flies were collected (although they had both been in laboratory culture for > 30 generations), the relative robustness of their immune responses to particular pathogens, or endemic/mutually beneficial host-parasite stability. These hypotheses are possible because they are based on documented evidence for the influence of environmental and host factors on extracellular gut bacteria and a broad range of somatic host functions in *D. melanogaster*^35,36^. Furthermore, common chronic, noninvasive associations with particular bacterial lineages are often beneficial, or even required, for the development and reproduction of hosts^37^. Beneficial effects include developmental interactions that prime the immune system and improve tolerance to thermal stress; these benefits might account for the differences we have observed in *C. rosa s.s* and *C. quilicii* which are geographically and thermally divergent.

Our transcriptomic analyses of transcripts revealed a greater investment in physiological processes associated with immunity, energy production, cell proliferation, insecticide resistance and degradation of the fruit metabolites in male *C. quilicii* compared with male *C. rosa s.s*. Given that our microbial analysis (above) revealed the presence of various bacteria within the guts of *C. quilicii*, it is possible that the immune responses observed in *C. quilicii* might be in response to these bacteria which could, in turn, reflect the relative abundance of these bacteria in the environmental biota, including within host plants. Higher demand for energy and proliferation in *C. quilicii* than in *C. rosa s.s*., suggests a higher intrinsic rate of growth and thus fitness. The presence of insecticide resistance genes shows that *C. quilicii* also has enhanced resistance to insecticides (and probably other xenobiotics) and/or it is under constant selection pressure for insecticide resistance. In contrast, *C. rosa s.s* harbours viral transcripts that may again reflect the abundance of these viruses in their environmental biota and/or a weaker immunity against viruses. In addition to the differences observed in male flies, comparisons of female *C. quilicii* and *C. rosa s.s* also revealed upregulation of pathways and genes essentially associated with reproduction and proliferation, but also with redox which is usually associated with responses to stress. Additionally, the presence of viral transcripts in female *C. rosa s.s* similar to those in male *C. rosa s.s* suggests either a potential environmental source for the viruses or their vertical transmission. Despite the discernible differences drawn from this study on the two sibling species in gene expression levels and microbiota of the mid-gut tissues and contents, the study is based on only laboratory colonies. Consequently, colony reared insects can only provide limited, but acceptable insight on phenotype of the flies in their natural habitats that should later be validated downstream. Therefore, the differentially expressed genes can be used as diagnostic markers for the two sibling species. Further studies with wild populations should be undertaken.

## Conclusion

In conclusion, this study identified potential natural gut microbes and host transcriptional profiles that could be used to define and differentiate between the sibling species, *C. rosa s.s*. and *C. quilicii*. We identified stable, free-living bacterial species (and others) in *C. quilicii* with potential for stable proliferation throughout the life cycle of the fruit fly. In *C. quilicii* we also identified greater investment in several physiological processes that distinguished this species from *C. rosa s.s*. which, in contrast, harboured several viruses that were not present in *C. quilicii*. Whether the extracellular gut microbiome we identified in *C. quilicii* are responsible for a potential reduction in reproductive fitness remains to be determined. Recent findings suggest that horizontally transmitted extracellular gut *Acetobacter* species can influence the *D. melanogaster* germ line by enhancing oogenesis linked to aldehyde dehydrogenase in the host^22^.

## Material and Methods

### Test insects

The *C. rosa s.s*. colony used in this study was established from individuals collected from guava fruits from Kibarani, Msambweni district (S 04°19'62.8”; E 039°32'41.1”; 34 m a. s. l), a coastal region of Kenya. The flies were reared in Plexiglass cages (65 cm × 35 cm × 65 cm) and the colony maintained at 28 ± 1 °C, 50 ± 8% RH and photoperiod of L12: D12. The *C. quilicii* colony was established from individuals collected from mango fruits from Kithoka, a highland region in Imenti North district (N 00°05'58.9”; E 037°40'39.5”; 1,425 m a. s. l) of the central region in Kenya. The *C*. *quilicii* was reared in Plexiglass cages and the colony maintained at at 23 ± 1 °C, 65 ± 5% RH and photoperiod of L12: D12. Both species were reared in a sterile environment and on carrot-based artificial diets^38^ with no antibiotic, in the *icipe* animal rearing and quarantine unit, Nairobi, Kenya. At the time of the bioassay, the *C. quilicii* colony was at the 59^th^ generation and the *C. rosa s.s*. colony was at the 36^th^ generation. Noteworthy, the establishment of the two colonies was initiated concurrently, however, the *C. rosa s. s*. colony took way much longer to be established at the *icipe* laboratories because the species is adapted to lowland temperate conditions. Because of this, the reproduction rates of *C. quilicii* was way higher and faster facilitating more generations as compared to its counterpart *C. rosa s. s*. which was slow.

### Extraction of *C. rosa s.s*. and *C. quilicii* RNA

Experimental insects were collected two days post emergence and maintained exclusively on water. The entire gut and its contents were dissected from individual male and female *C. rosa s.s* and *C. quilicii* in 1X phosphate buffered saline (PBS) (pH 8.0) under a Leica (EZ4HD) stereoscope (n = 100 for each sex of each species); the guts for each sex/ species combination were then pooled and stored in liquid nitrogen until required for downstream analyses. Total RNA was extracted using the Isolate II RNA Mini Kit (Bioline, London, UK), following the manufacturer’s instructions, and the resulting RNA immediately stored at −80 °C. RNA yield was determined using a Nanodrop 2000/2000c Spectrophotometer (Thermo Fisher Scientific, Waltham, Massachusetts, USA), and the size and preliminary integrity of the RNA determined by gel electrophoresis on 1.2% non-denaturing agarose followed by ethidium bromide staining. Visualization and documentation of the gel image was done using a KETA GL imaging system (Wealtec Corp, Meadowvale Way Sparks, Nevada, USA). The RNA (50 uL) was subsequently lyophilized and preserved in RNAstable tubes (Biomatrica, Inc, USA) as per the manufacturer’s instructions until the RNA was required for NGS.

### RNA sequencing of the *C. rosa s.s*. and *C. quilicii* transcriptomes

The integrity of RNA from each sample was assessed individually using a Bioanalyzer (Agilent Technologies, USA), and cDNA was generated using an Illumina *TruSeq RNA* Sample Preparation Kit *(*Illumina, Hayward, CA, USA). This technique is based on rRNA depletion and not polyA + selection which means that both prokaryotic and eukaryotic transcripts are retained in the libraries. Forward-Reverse (FR) strand-specific libraries were prepared from each sample and were sequenced by Macrogen Inc. (Seoul, South Korea) using an Illumina HiSeq. 2000 (Illumina, Hayward, CA, USA) platform with two 101 nt paired-end reads. Low quality reads, reads with less than 101 base pairs, and adapter sequences were removed using Illumina build software (Illumina, Hayward, CA, USA) as part of the sequence clean-up. This generated final fastq formatted raw data for each library. Overall, eight fastq files were generated, a pair each for each sibling species and sex (i.e. (*C. rosa s.s* and *C. quilicii*, male or female) × 2).

### Identification and validation of transcripts that were differentially expressed (DE) in *C. rosa s.s*. and *C. quilicii*

The quality of the RNA sequence reads in each file was assessed using FastQC software (http://www.bioinformatics.babraham.ac.uk/projects/fastqc/) and the high quality data used to clean reads using fastx quality trimmer (http://hannonlab.cshl.edu/fastx_toolkit/) software. Since both the *C. rosa s.s*. and *C. quilicii* reference genomes were not available (unpublished), we could not employ established genome-guided and gene-set based strategies to evaluate the expression profiles of the transcripts. We thus employed a *de novo* transcript assembly-based strategy to generate the transcripts from the transcriptomes. We then separated them into microbial and host transcripts and then identified which transcripts (microbial or host) were differentially expressed in the different libraries. Finally, we established which metabolic pathways were predominant and functionally associated with the differential transcript expression profiles using geneset enrichment analysis. Briefly, all the transcriptomes were combined, assembled *de novo* into transcripts and the quality of the assembled transcripts assessed using the short reads Trinity assembly program^24,39^. We isolated the longest transcripts (most representative of the respective genes) with open reading frames that could be translated into peptides that were at least 100 amino acids long using the TransDecoder program^24^. To separate the microbial transcripts from the host transcripts amongst these long transcript sets, we mapped the transcripts using the OrthoMCL^25^ ortholog against the orthologs (OrthoMCL) database var 5^26^. All sources of known transcripts from available microbe databases were identified using OrthoMCL. This separated the transcripts into those that represented microbial species and those that were host transcripts based on differences in their codon usage bias. The database contained 810, 686 ortholog groups clustered from 1, 398, 546 proteins obtained from 150 genomes. The genomes consisted of four Amoebozoan, six Firmicutes, nine Euglenozoan, 11 Viridiplantae, 15 Alveolates, 16 Archaea, 19 Proteobacteria, 24 fungi and 29 Metazoan taxonomic groups, in addition to six and 11 other eukaryote and bacteria taxonomic groups respectively^27^. Furthermore, the identification of orthologous groups in prokaryotic genomes has permitted cross-referencing of genes from multiple species, facilitating genome annotation, protein family classification and studies on bacterial evolution^40–43^. The transcripts were then queried against the BLAST non-redundant database which served to; (i) confirm the microbe ID’s and (ii) identified putative ID’s of the novel transcripts that could not be identified in the microbial database. To identify transcriptional expression profiles for both the microbial transcripts and the host transcripts that were differentially expressed between the libraries (sibling species), the cleaned reads from individual transcriptomes in the microbial or host transcript sets were individually mapped using CLC genomic workbench version 9.5 (CLC Bio, Aarhus, Denmark). The resultant mapping read counts for each library on each transcript set were used to determine differential expression of the respective microbial or host (male or female) transcripts using edgeR analysis^44,45^ software. To minimize detection of false positives for differential expression, a conservative regime was adopted against both libraries. Within this regime, we considered transcripts to be differentially expressed (DE) between treatments if: 1) they had at least a two-fold difference in expression level; 2) the false detection rate (FDR) was corrected p < 0.05; 3) they were supported by at least 100 read mappings in either categories (i.e. in *C. rosa s.s*, *C. quilicii* or microbial transcripts); and 4) they had five CPM (Counts Per Million). Fold changes as a ratio of CPM values between microbial or host transcripts from the *C. rosa s.s* and *C. quilicii* libraries were defined. Moreover, the metabolic pathways and networks that underwent differential functional enrichment (expression) between the two hosts (*C. rosa s.s* vs. *C. quilicii*) or between genders were identified using secondary canonical gene set enrichment analysis (GSEA) approaches within the WEB-based GEne SeT AnaLysis Toolkit (WebGestalt)^46^.

### Validation of transcriptome expression profiles using qRT-PCR

Since our data were derived from a single transcriptome for each treatment (i.e. species and gender), we employed a quantitative reverse transcription Polymerase Chain Reaction (qRT-PCR) technique as an independent tool to determine whether our RNA-Seq analysis results could potentially be independently replicated; we compared fold changes in randomly selected DE host transcripts that were achieved using the two approaches. This strategy has been employed successfully for validation of RNA-seq expression levels from single transcriptomes in other studies^47–50^. We did the validation using eight independent biological replicates (n = 8 for each of the different species and gender combinations) obtained from dissected flies generated under similar experimental conditions to those described for the transcriptome samples. Briefly, we prepared cDNAs (from 1 µg Total RNA) from the midgut of each replicate using High Capacity cDNA reverse transcription kit (Applied Biosystems, Carlsbad, CA) according to the manufacturer’s protocol. Quantitative RT-PCR was done on each replicate and specifically on 11 randomly selected DE genes using gene-specific primers (as detailed in Supplementary Information S1). The reaction mix consisted of 3 µg cDNA template that was amplified from each biological replicate; there were three independent technical replicates each with 7.5 µL of Fast SYBR^®^ Green master mix (Applied Biosystems, Carlsbad, CA) and 0.5 picomoles of each of the specific primers for the various genes of interest. Reactions were made in the Strategene MX3005P real time qPCR machine (Agilent Technologies, California, USA). All qRT-PCR results were normalized to two genes that were expressed by Bestkeeper^51^ in the two sibling species, which were also quantified from each biological replicate. REST software^52^ was used for pairwise gene expression analysis, including an internal multiple-tests correction. Fold changes in transcript expression levels were established by comparing levels of expression of the transcripts in the midguts of *C. rosa s.s* with those in the midguts of *C. quilicii*. We conducted Pearson correlation analysis of the fold changes that were obtained from qRT-PCR with those that had been obtained previously from the RNA-seq data to determine the validity of our transcriptomes. Separate validation of the microbial transcriptomes was not performed since they were inherently intertwined with the host transcriptomes and generally had lower expression levels which would be technically difficult to detect using qRT-PCR.

### Ethics approval

All insect rearing, handling and experiments were performed using standard operating procedures at the ICIPE Animal Rearing and Quarantine Unit as approved by the National Commission of Science, Technology and Innovations, Kenya.

## Supplementary information

Supplementary Info

Table S1

Table S2

## Data Availability

The datasets generated and/or analysed during the current study are available from the corresponding author upon request and will also be made available through open source platforms.
