# Supplementary material for: Species-specific transcriptional profiles of the gut and gut microbiome of Ceratitis quilicii and Ceratitis rosa sensu stricto
Source: Sci Rep. 2019 Dec 4;9:18355. doi: 10.1038/s41598-019-54989-z (PMC6892911; doi:10.1038/s41598-019-54989-z)

Species-specific transcriptional profiles of the gut and gut microbiome of *Ceratitis quilicii* and *Ceratitis rosa sensu stricto*

Fathiya M. Khamis[1*], Paul O. Mireji[2,3], Fidelis L.O. Ombura[1], Anna R. Malacrida[5], Erick O. Awuoche[2,4], Martin Rono[3], Samira A. Mohamed[1], Chrysantus M. Tanga[1] and Sunday Ekesi[1]

[1]International Centre of Insect Physiology and Ecology, P.O. Box 30772-00100, Nairobi, Kenya

[2] Biotechnology Research Institute, Kenya Agricultural and Livestock Research Organization, P.O. Box 362-00902, Kikuyu, Kenya

[3]Centre for Geographic Medicine Research Coast, Kenya Medical Research Institute, P.O. Box 428, Kilifi, Kenya

[4]Department of Agriculture, School of Agriculture and Food Science, Meru University of Science and Technology, P.O. Box 972, Meru, Kenya

[5]Department of Biology and Biotechnology, Universita degli Studi di Pavia, Corso Strada Nuova, 65, 27100 Pavia, Italy

fkhamis@icipe.org, mireji.paul@gmail.com, lombura@icipe.org, malacrid@unipv.it, otieno43@gmail.com, MRono@kemri-wellcome.org, sfaris@icipe.org, ctanga@icipe.org, sekesi@icipe.org

*Author for correspondence: Fathiya M Khamis, Fax: +254 (20) 8632001, E-mail: fkhamis@icipe.org

SI Guide

Supplementary Information

Figure S1: Profile of transcripts that were differentially expressed between *C. rosa s.s* and *C. quilicii* (combined sexes).

Text S1: List of primers used to validate male and female *Ceratitis rosa s.s* and *Ceratitis quilicii* transcriptomes.

Text S2: Validation of male and female *Ceratitis rosa s.s* and *Ceratitis quilicii* transcriptomes.

Supplementary Dataset

Table S1: Transcripts that were differentially expressed between male *C. rosa s.s* and male *C. quilicii*.

Table S2: Transcripts that were differentially expressed between female *C. rosa s.s* and female *C. quilicii*.

Figure S1: Profile of transcripts that were differentially expressed between *C. rosa s.s* and *C. quilicii* (combined sexes).

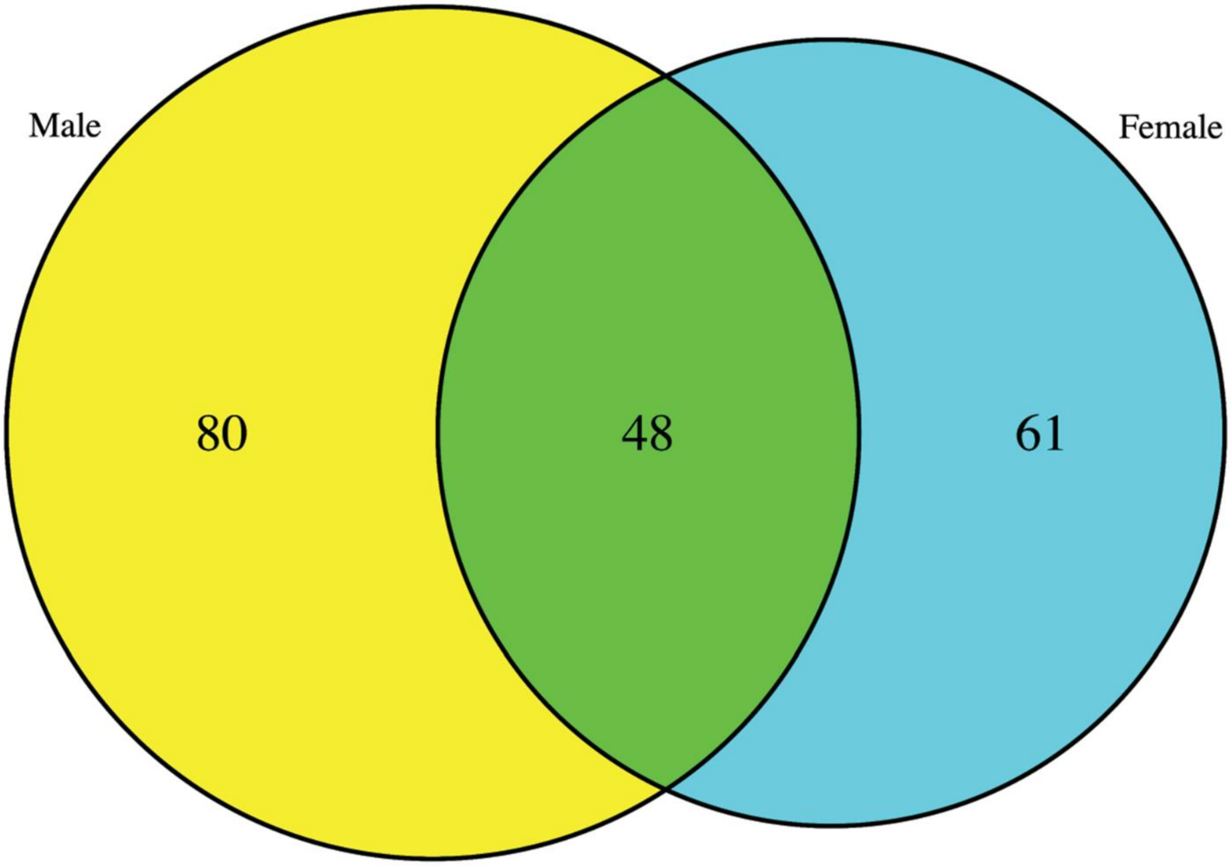

Text S1: List of primers used to validate male and female *Ceratitis rosa s.s* and *Ceratitis quilicii* transcriptomes.

Name	Sequence 5' - 3'	Size (bp)	Annealing temp (°C)	Target
C.r_8248_Fwd	TGTCAGAGTTGTCTCGTCGTC	524	57.7	Internal control, males only
C.r_8248_Rev	TAGCTCTTCTGTACTCAGGTCC			
C.r_4990_Fwd	AATGTGCGTTATGTCGCTGC	333	57.7	
C.r_4990_Rev	AGTGTCAATATCCTCAACTTTATGC			
C.r_8216_Fwd	TTTCTTCTGGGTGGAGTAGGCG	110	53.1	
C.r_8216_Rev	ACGAAACGCCTGAAGCAATCT			
C.r_7869_Fwd	GCGTTGCTCCTGGAACTTCT	336	56.1	Internal control, females only
C.r_7869_Rev	CGTATATTAAGGGCACTCCAACTG			
C.r_11636_Fwd	GAAACATCACAGCGTTAGCAC	144	56.1	
C.r_11636_Rev	ATACGCCGTTGACGTTCCAT			
C.r_12500_Fwd	CTTGGACAACGCTTGTGGTG	597	59.3	
C.r_12500_Rev	CCTCGACTGCTTCTACTAGCG			
C.r_12449_Fwd	GTTTGAACGGAGCCCCC	724	64.7	
C.r_12449_Rev	GGTTGGCCTGTTAAGCCGAT			
C.r_10610_Fwd	AACGCTTCTTCGTGCTGGCT	433	60.1	Both males and females
C.r_10610_Rev	CTCTCCTCAACTGGCAACACA			
C.r_12399_Fwd	AATGAGGTCACCGCAGAAGG	302	63	
C.r_12399_Rev	GTCCAGACATTCCGCTTCCA			
C.r_12610_Fwd	AGGACAAGGCGGTCAAAAGT	283	54	
C.r_12610_Rev	GCCTTGAAGACCGTAAGGGA			
C.r_4115_Fwd	CGCTGATCCATGACAAGGCA	526	59.1	
C.r_4115_Rev	TTCTCGGTAGCATACCAGTGT			
C.r_6247_Fwd	TGATGCCGAAGTGTTCCCAA	183	57.9	
C.r_6247_Rev	TTGTCCATCTTAGCGGCCTT			
C.r_7786_Fwd	CGAAAACCCCGGTAGGAACA	107	55	
C.r_7786_Rev	GTCACGTACTGCCCCTGAAA			
C.r_8723_Fwd	AAGTGGTCGCGATACACGAG	106	53	
C.r_8723_Rev	ACGCAACACTGCAAAAGCAA			
C.r_8724_Fwd	ATTCAGCCCAATCCCTGACG	477	55	
C.r_8724_Rev	GCTGGTTGCATTCTTGGAGC			
C.r_9667_Fwd	CCACCACCGCAATAACTGGT	140	57.8	
C.r_9667_Rev	GGTGCGATAGTTGGTGTCCA			
C.r_10341_Fwd	CGCTGGGCACAGAAATACG	380	58	Males only
C.r_10341_Rev	ATGGCGGCCTCGCATATAAT			
C.r_12300_Fwd	TCAATGGGACACCTGCTCAC	125	54.2	
C.r_12300_Rev	GTGCTGCTCGTTTCAGTGTG			
C.r_12563_Fwd	TTGTAACTACGCCGATGCCA	284	53	

C.r_12563_Rev	TAGCGTCGAGTGTAGCGTTG			
C.r_12556_Fwd	TAGTGATGGGGCCGGTAATG	581	61.8	
C.r_12556_Rev	CTCCTCATTGCGTGCCTGC			
C.r_12564_Fwd	ATCACACTGGAGCCTTCGAC	140	64	
C.r_12564_Rev	CGCAGGCTCGAAGACCA			
C.r_5556_Fwd	TGGATCGTCGAAACCGAAAG	343	54.7	
C.r_5556_Rev	TTCGGGTAAATCGGTGCTGTA			
C.r_5612_Fwd	GTGCCGCCTTACCCATTCTA	233	54.2	
C.r_5612_Rev	TTGTCTGCCGCTCCATTCAT			
C.r_6163_Fwd	TCAGAAGCACGTGGAATGCT	238	61.8	
C.r_6163_Rev	GGGGTGGTAACGCGAATACT			
C.r_7507_Fwd	ACAATGTAATCGGCGGCAAC	232	53	
C.r_7507_Rev	TGTATGGCTTCGGTTGTGGT			
C.r_12895_Fwd	CCAGCCACCGTCCATACTAAA	223	59.3	Females only
C.r_12895_Rev	CACCCCAAGTCTTCGCTTCT			
C.r_2018_Fwd	GCGGAACTCCAATCTATGCCA	249	60.9	
C.r_2018_Rev	GCCAAAGTTGGGCTGTTGTT			
C.r_1404_Fwd	AACCAGATTCCCGCCTACAC	306	56.1	
C.r_1404_Rev	GGGTTTAGATTGTTGGCCGTT			
C.r_7470_Fwd	TGGATCGTTTGACTACAGCCAT	126	59.3	
C.r_7470_Rev	CCTGGAAACCGTCCTTACCC			
C.r_4979_Fwd	CCGAAGCAATGCAGATTTCG	164	53.1	
C.r_4979_Rev	CGTCAACCTTAAGCACAATGG			
C.r_1802_Fwd	TCTGCCATCGCGTCGTTT	247	57.7	
C.r_1802_Rev	TCTCAAGCGCTGTTCTTCCA			
C.r_4115_Fwd	CGCTGATCCATGACAAGGCA	526	54.4	
C.r_4115_Rev	TTCTCGGTAGCATACCAGTGT			
C.r_6247_Fwd	TGATGCCGAAGTGTTCCCAA	183	57.7	
C.r_6247_Rev	TTGTCCATCTTAGCGGCCTT			

Text S2: Validation of male and female *Ceratitis rosa s.s* and *Ceratitis quilicii* transcriptomes.

Validation of **MALE** *Ceratitis rosa s.s* and *Ceratitis quilicii*, sibling species RNA-seq results with qPCR. The expression values (log_2 ratios) for eleven genes are plotted against qPCR values (log_2 ratios). The Pearson correlation coefficient (**R=0. 814611751)**and Goodness fit, **R^2 = 0.66359** obtained are quite high indicating high correlation. These results indicate that the qPCR correctly validates the male *Ceratitis* RNA-seq data.

Feature ID	Fold change in qPCR	Log2 fold change qPCR	RNA-seq Fold change (Log2)
C.r_12564	-1.21419	-0.28	-0.19755
C.r_12399	-1.17589	-0.23375	-2.27165
C.r_7786	2.77022	1.47	3.83916
C.r_6247	3.34905	1.74375	2.53053
C.r_4115	3.72567	1.8975	2.67972
C.r_9667	4.92458	2.3	3.67231
C.r_10610	6.72135	2.74875	2.6946
C.r_7507	8.44882	3.07875	4.06499
C.r_5612	10.43783	3.38375	4.08097
C.r_5556	13.10913	3.7125	4.23187
C.r_12610	14.96738	3.90375	2.70732

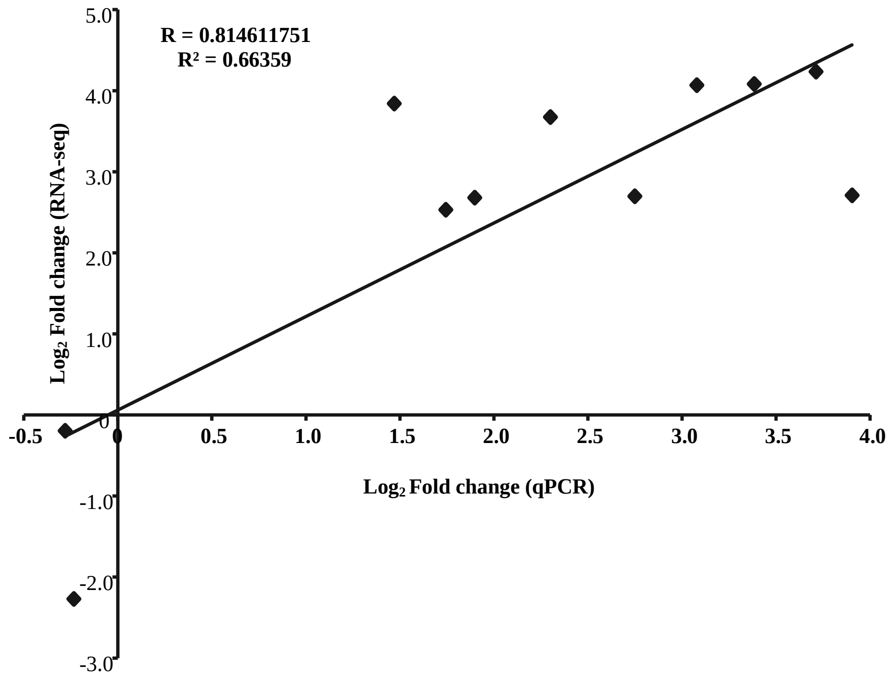

Validation of **FEMALE** *Ceratitis rosa s.s* and *Ceratitis quilicii*, sibling species RNA-seq results with qPCR. The expression values (log_2 ratios) for eleven genes are plotted against qPCR values (log_2 ratios). The Pearson correlation coefficient (**R= 0.933804772**) and Goodness fit, **R^2 = 0.87199** obtained are quite high indicating high correlation. These results indicate that the qPCR correctly validates the female *Ceratitis* RNA-seq data.

**Feature ID**	**Fold change in qPCR**	**Log_2 fold change qPCR**	**RNA-seq Fold change (Log_2)**
C.r_12399	-90.58812	-6.50125	-5.12828
C.r_2018	-2.94598	-1.55875	-3.13308
C.r_1404	-26.1502	-4.70875	-2.37815
C.r_12895	-1.17895	-0.2375	0.08821
C.r_7470	-9.62976	-3.2675	-2.91521
C.r_4979	-7.86256	-2.975	-3.14058
C.r_10610	2.66297	1.41304	2.75778
C.r_12610	12.87276	3.68625	2.62901
C.r_1802	8.38319	3.0675	2.51119
C.r_4115	1.93858	0.955	2.33294
C.r_6247	3.21262	1.68375	2.49114

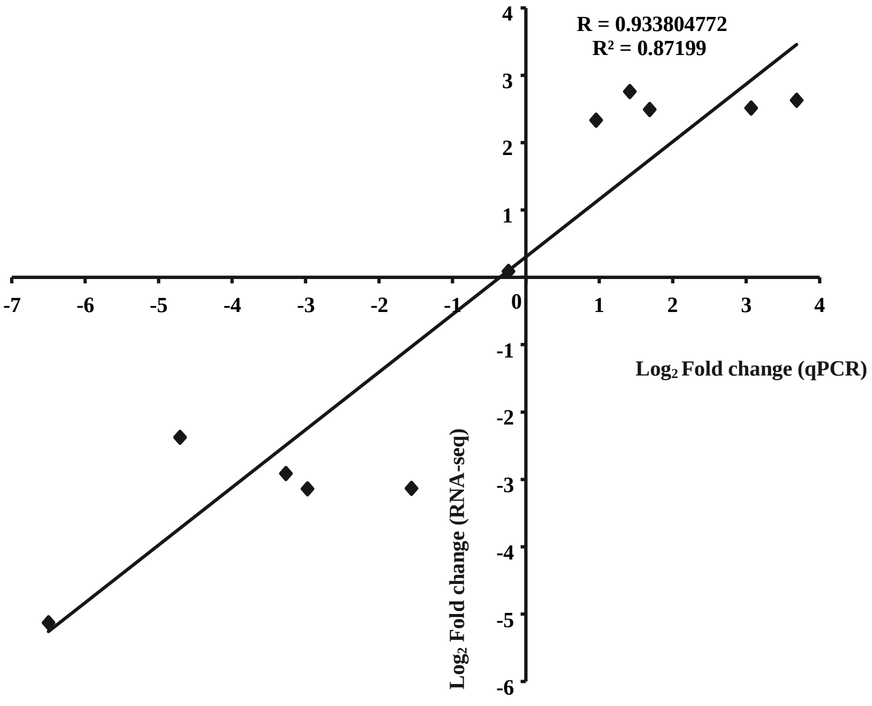

Supplement: Supplementary file 1 — Supplementary Info [file 41598_2019_54989_MOESM1_ESM.pdf]
